# *In vitro* enhancement of dendritic cell-mediated anti-glioma immune response by graphene oxide

**DOI:** 10.1186/1556-276X-9-311

**Published:** 2014-06-20

**Authors:** Wei Wang, Zhongjun Li, Jinhong Duan, Chen Wang, Ying Fang, Xian-Da Yang

**Affiliations:** 1 Peking University People’s Hospital, Peking University Hepatology Institute, Beijing 100044, China; 2 Institute of Basic Medical Sciences, Chinese Academy of Medical Sciences and Peking Union Medical College, Beijing 100005, China; 3 National Center for Nanoscience and Technology, Beijing 100190, China

**Keywords:** Glioma, Dendritic cell, DC, Graphene oxide, GO, Immunotherapy

## Abstract

Malignant glioma has extremely poor prognosis despite combination treatments with surgery, radiation, and chemotherapy. Dendritic cell (DC)-based immunotherapy may potentially serve as an adjuvant treatment of glioma, but its efficacy generally needs further improvement. Here we explored whether graphene oxide (GO) nanosheets could modulate the DC-mediated anti-glioma immune response *in vitro*, using the T98G human glioma cell line as the study model. Pulsing DCs with a glioma peptide antigen (Ag) generated a limited anti-glioma response compared to un-pulsed DCs. Pulsing DCs with GO alone failed to produce obvious immune modulation effects. However, stimulating DCs with a mixture of GO and Ag (GO-Ag) significantly enhanced the anti-glioma immune reaction (*p* < 0.05). The secretion of interferon gamma (IFN-γ) by the lymphocytes was also markedly boosted by GO-Ag. Additionally, the anti-glioma immune response induced by GO-Ag appeared to be target-specific. Furthermore, at the concentration used in this study, GO exhibited a negligible effect on the viability of the DCs. These results suggested that GO might have potential utility for boosting a DC-mediated anti-glioma immune response.

## Background

Malignant glioma is the most common primary brain tumor with grim prognosis. Current therapies, including surgery, radiation therapy, and chemotherapy, present limited efficacies for treating malignant glioma [1,2]. Local control of the tumor is difficult in more than 80% of cases, because glioma cells infiltrate the surrounding tissues with high capabilities of migration and invasion. Even with intensive treatment, gliomas frequently recur due to the growth of residual diseases beyond the surgical resection margins [3,4]. During the past 30 years, little improvement in survival time has been achieved for patients with high-grade (grades III and IV) glioma, and long-term survival is rare [5]. This situation has stimulated a strong interest in developing novel therapies for malignant and recurrent gliomas.

Dendritic cell (DC)-based immunotherapy represents a promising approach for development of novel therapies against malignant glioma. DCs play a central role in generating a specific immune reaction to antigens, which generally need to be ingested, processed, and presented by DCs, before triggering a B cell- or T cell-mediated response. This key immune mechanism has been utilized in designing DC-based anti-cancer immunotherapy, whereby a patient's DCs are expanded with *in vitro* culture, stimulated with tumor antigen, and injected back to the body to elicit anti-cancer immune reactions [6]. DC-based immunotherapy generated promising results in some early-stage clinical trials [7-10]. Yu et al. reported that vaccination with DCs pulsed by tumor lysate was safe and not associated with any evidence of autoimmune disease [7]. Moreover, the median survival time of the treated patients was prolonged, suggesting that DC-based immunotherapy had the potential to improve the prognosis of glioma. Nonetheless, the immunogenicity of glioma antigens is generally weak, and novel technology is urgently needed to boost the immune reaction induced by glioma antigens.

Graphene oxide (GO), a nanomaterial first reported in 2004 [11], has attracted much attention because of its application prospective in biomedical fields [12-15]. GO has relatively large two-dimensional surfaces that can absorb various bioactive molecules [16,17]. GO also possesses excellent capability for traversing the cell membrane and facilitating the cellular uptake of both small and macro-molecules, with good biocompatibility, limited cytotoxicity, and high loading ratio [12-14,17-19]. GO has been evaluated as potential vehicles for the intracellular delivery of various bioactive molecules, including genes and anti-cancer drugs [12-14,17,18]. So far, however, no attempt has been reported in literature to use GO for modulation of anti-cancer immunity. Given the excellent features of GO as a transporter of molecules across the cell membrane [19], it will be interesting to study whether GO can carry more glioma antigens into DCs and modulate the DC-mediated anti-glioma immune reaction. In this work, we explored whether GO would affect the immunogenicity of a known glioma peptide antigen (Ag). The peptide antigen is from the protein survivin, which is commonly expressed in human and murine malignant gliomas [20-22]. We found that a mixture of GO and Ag (GO-Ag) induced a more potent DC-mediated anti-glioma immune reaction *in vitro*. The results indicate that GO-based nanotechnology may have a prospective role in development of more efficacious anti-cancer immunotherapies.

## Methods

### Cell lines and reagents

T98G is a glioblastoma cell line with documented overexpression of survivin, with epitopes associated with human leukocyte antigen (HLA)-A2 [23]. T98G cells were cultured in DMEM (Gibco, Life Technologies, Carlsbad, CA, USA) supplemented with 10% heat-inactivated fetal bovine serum (FBS; HyClone, Thermo Fisher Scientific, Waltham, MA, USA). The HLA-A2-positive T2 cell line was cultured in RPMI 1640 (Gibco, Life Technologies, Carlsbad, CA, USA) supplemented with 10% FBS. The two cell lines were maintained at 37°C in 5% CO_2_ with media replaced two or three times per week. Recombinant human granulocyte macrophage colony-stimulating factor (rhGM-CSF) was purchased from Beijing Medical University United Pharmaceutical Co., Ltd. (Beijing, China). Recombinant human interleukin (rhIL)-4 and tumor necrosis factor (TNF)-alpha; fluorescein isothiocyanate (FITC) mouse anti-human CD83, CD86, and HLA-DR; and their respective isotype controls were purchased from BD Pharmingen (San Jose, CA, USA).

### Preparation and characterization of GO

GO was prepared by a modified Hummer's method [24]. Briefly, powder graphite (1,500 mesh, 10 g) and KMnO_4_ (120 g) were slowly mixed with concentrated H_2_SO_4_ (98%, 1 L) while maintaining vigorous agitation in an ice bath. The ice bath was replaced with a water bath, and the ingredients were agitated overnight. Distilled water (2 L) was carefully and slowly added to the complex. Next, 30% H_2_O_2_ was added to remove the residual potassium permanganate when the mixture showed a gray-black color. The bright yellow mixture was filtered and washed with 10% HCl solution (2 L) twice. The filter cake was dispersed in distilled water and centrifuged repeatedly for thorough washing. Finally, the paste at the bottom of the centrifuge tube was carefully collected and dispersed in distilled water as the stock solution (about 2 mg/mL). In order to obtain nanosized GO, the stock solution was probe-sonicated at 500 W for 2 h and the GO nanosheets were separated via centrifugation (50,000 *g*, 1 h). The deposit was then collected and dispersed as the nanosized GO solution.

Characterization of GO nanosheets was achieved with atomic force microscopy. The morphology of the nanosheets was revealed using Dimension 3100 (Veeco, Plainview, NY, USA) atomic force microscope with a typical silicon tip (Olympus, Shinjuku-ku, Japan) in tapping mode.

### Peptides

The survivin peptide ELTLGEFLKL is a HLA-A2-restricted peptide, which has been described previously to induce HLA-A2-restricted T cell reactions [25,26]. The control peptide APDTRPAPG is also a HLA-A2-binding peptide and thus can be presented by HLA-A2. The peptides were synthesized by SBS Genetech Co., Ltd. (Beijing, China), and the purity was more than 95%. The peptides were dissolved in DMSO (10 mg/mL) as the stock solution and stored at -80°C. To prepare the working solution, the stock solution was diluted in sterile deionized water (1 mg/mL) and then stored at -20°C.

### Loading peptide onto GO and evaluation of the loading capacity

Loading peptides onto GO was accomplished by sonicating the GO suspension (10 μg/mL) with the peptide solution at an equal volume ratio for 30 min. The complex was shaken on a shaker at room temperature for 1 h. A light-brown-colored homogeneous suspension was formed and ready for further application. Peptide solution or GO suspension alone was also prepared in a similar way to serve as controls. To determine the loading rate of the peptide onto GO, the mixtures of GO and peptide with different peptide/GO feed ratios (ranging from 0.2 to 12.5) were prepared and centrifuged at 12,000 rpm for 30 min. The deposits were further washed with water and centrifuged twice. The supernatants were collected, and the amounts of peptides in the supernatants were measured using a standard bicinchoninic acid (BCA) assay. The amount of complexed peptide was calculated after deducting the amount of peptide in the supernatant.

### HLA typing

Peripheral blood was obtained from healthy human donors. Genomic DNA was extracted and purified from whole blood or T98G cells using a DNA extraction kit (Gene Tech, Shanghai, China) according to the manufacturer's protocol. DNA typing for HLA-A2 alleles was determined by PCR using sequence-specific primers and sequence-based typing as reported before [27]. The primers (Invitrogen, Life Technologies, Carlsbad, CA, USA) were as follows:

• Forward primer: 5′-CACTCCTCGTCCCCAGGCTGT-3′

• Reverse primer: 5′-CGTGGCCCCTGGTACCCGT-3′

The thermal profile was 94°C for 10 min, followed by 33 cycles of 94°C for 50 s, 66°C for 50 s, and 72°C for 50 s, and then 72°C for 10 min.

### DC culturing and antigen pulsing

Peripheral blood mononuclear cells (PBMCs) of HLA-A2-positive healthy human donors were isolated by standard Ficoll gradient centrifugation of heparinized blood, washed with D-Hank's solution, and divided into two parts. One half of PBMCs were used for DC culture, and the other half were frozen until they were used as effector cell production in later experiments. For DC culturing, PBMCs were suspended in RPMI 1640 with 10% FBS and adhered in culture flasks for 2 to 4 h at 37°C in a 5% CO_2_ incubator. Non-adherent cells were removed by washing, and the remaining adherent cells were cultured in RPMI 1640 with 10% FBS supplemented with recombinant human GM-CSF (1,000 IU/mL) and IL-4 (20 ng/mL) for 5 to 6 days. Then, immature DCs were harvested and pulsed with GO (0.1 μg/mL), Ag (1, 5, or 10 μg/mL), or GO-Ag complex (GO-Ag; 1, 5, or 10 μg/mL) for 2 h. In the control group, DCs were pulsed with D-Hank's buffer only. After that, DCs were washed with D-Hank's buffer and harvested for further studies.

### Immune response against glioma cells

The *in vitro* evaluation of DC-mediated anti-tumor response was performed as previously described [28]. Briefly, GO-, Ag-, or GO-Ag-pulsed DCs (1 × 10^5^/well) were co-cultured with syngeneic PBMCs (2 × 10^6^/well) in 24-well plates for 5 days in the presence of TNF-alpha (20 ng/mL, BD Pharmingen, San Jose, CA, USA). On day 3, the culture media were replaced and rhIL-2 (10 IU/mL) was added. After 5 days, PBMCs were collected as effector cells for anti-tumor immune response study. Firstly, T98G cells (target cells) were added to 96-well U-bottom plates at a density of 3 to 5 × 10^3^/well for 2 to 4 h to become adherent. Then, the effector cells and target T98G cells were mixed in the 96 wells at an effector-to-target ratio (E:T) ratio of 20:1. The background control wells contained only medium, while the positive control contained only the target cells and medium without the effector cells. Six wells were used for each group. After co-incubation with target cells in a 5% CO_2_ incubator at 37°C for 2 to 3 days, PBMCs were removed and the plates were washed twice with D-Hank's solution. The tumor inhibition rate was then measured using a standard MTS assay according to the manufacturer's (Promega, Madison, WI, USA) instruction (*n* = 6). An MTS/PMS mixture of 20 μL was added into each well of the 96-well plate, followed by incubation for about 2 h at 37°C. When the color of the culture media turned brown, the plates were measured for light absorption by an enzyme-linked immunosorbent assay (ELISA) plate reader at 490 nm. The percentage of tumor growth inhibition was calculated according to the following equation (*A*_490_ indicates the light absorption at 490 nm):

$$\begin{array}{ll} \%\mathrm{Growth}\;\mathrm{inhibition}\;\mathrm{rate}= & \left[1-\frac{{A_{490}}_{{}_{\mathrm{Experimental}\;\mathrm{well}}}-{A_{490}}_{{}_{\mathrm{Background}}}}{{A_{490}}_{{}_{\mathrm{Positive}\;\mathrm{well}}}-{A_{490}}_{{}_{\mathrm{Background}}}}\right]\\ & \times 100 \end{array}$$

### ELISA for IFN-γ detection

DCs were pulsed with GO (0.1 μg/mL), Ag (5 μg/mL), or GO-Ag (5 μg/mL) for 2 h and washed by D-Hank's solution. Then, syngeneic PBMCs were added and incubated with DCs for 3 days. The supernatants of the culture were collected and measured for interferon gamma (IFN-γ) with an IFN-γ ELISA kit (Dakewe Biotech Company, Shenzhen, China) according to the manufacturer's protocol (*n* = 6).

### Peptide-specific immune response

Peptide-specific immune response study was evaluated using a non-radioactive cytotoxicity assay kit (Promega, Madison, WI, USA) and the HLA-A2-expressing T2 cell line. T2 is a hybrid B-T lymphoblastic cell line as a typical model system for studying class I antigen presentation and peptide-specific cytotoxicity study [29]. PBMCs were co-incubated with GO-Ag (5 μg/mL)-pulsed DCs for 5 days as described above. The PBMCs were washed and used as effector cells. T2 cells (2 × 10^5^ cells/well) were loaded with Ag (5 μg/mL) or the control peptide (5 μg/mL) overnight and washed to serve as target cells. The effector and target cells were then co-incubated at designated E:T ratios in 96-well plates for 4 h at 37°C in 5% CO_2_. The peptide-specific immune-mediated lysis of the cells was measured by testing lactate dehydrogenase (LDH) release in the supernatant per manufacturer's instruction (*n* = 6).

### Flow cytometric analysis

The phenotype of DCs after stimulation was assessed by studying the expression of cell surface markers. DCs were pulsed with GO (0.1 μg/mL), Ag (5 μg/mL), or GO-Ag (5 μg/mL) for 2 h, washed, and incubated overnight at 37°C in a 5% CO_2_ incubator. DCs were then collected and suspended in cold staining buffer (PBS containing 1% FCS, 0.1 mL) in microcentrifuge tubes. Afterwards, 20 μL of FITC-labeled anti-CD83, CD86, and HLA-DR monoclone antibodies (BD Pharmingen, San Jose, CA, USA) were added and incubated with DCs for 30 min at 4°C. The DCs were washed again with cold staining buffer for three times, and the cell surface markers were analyzed by flow cytometry.

### Cellular viability study

The influence of GO on DC viability was checked with a standard MTS cell viability assay according to the manufacturer's direction. Briefly, DCs were treated with GO (0.1 μg/mL) or D-Hank's solution in 24-well plates for 2 h at 37°C in 5% CO_2_, washed thoroughly, and then added into 96-well plates with a density of 1 × 10^4^/well. After 1, 4, and 24 h of incubation, the viability of DCs was evaluated with the MTS cell viability assay (*n* = 6).

### Statistical analysis

Statistical difference was determined by Student's *t* test, and a value of *p* < 0.05 was considered statistically significant.

## Results

**Figure 1:**
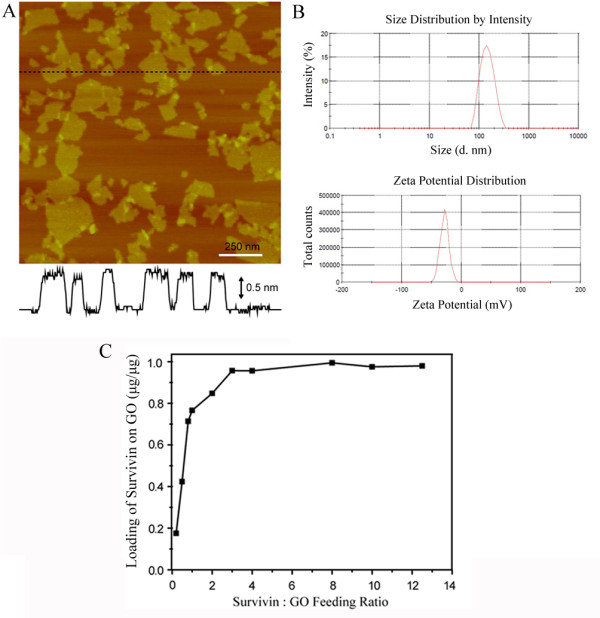
**Characterization of GO nanosheets and their antigen loading capability. (A)** AFM topographic image of nanosized GO sheets deposited on mica (top) and the height profile along the black line (bottom). Scale bar is 500 nm. **(B)** Distributions of size and zeta potential of GO. **(C)** Loading rates of Ag on GO at various peptide/GO feed ratios.

GO was prepared from natural graphite by a modified Hummer's method [24]. In order to get exfoliated single-layer nanosized GO, the GO solution was further processed and cracked by ultrasonication. The GO nanosheets were next collected via centrifugation at 50,000 g and dispersed in water as the stock solution. Atomic force microscopy (AFM) characterization (Figure 1A) provided morphological information of the GO nanosheets. The height profile showed that the thickness of GO nanosheets was around 1.1 nm (Figure 1A), indicating single-layer nanosheets. Moreover, the lateral size of GO nanosheets was about 60 to 360 nm, with an average dimension of 140 nm. The GO was negatively charged with an average zeta potential of -28 mV (Figure 1B). The GO solutions were used without further treatments in the following experiments.

To induce a specific anti-glioma immune response, DCs must be exposed to glioma antigens. The antigen used in the study was a peptide (ELTLGEFLKL, termed Ag) from the protein survivin, which is widely expressed in malignant gliomas [20-22]. Survivin is a member of the inhibitor of apoptosis (IAP) protein family, which can regulate two important cellular processes: it inhibits apoptosis and promotes cell proliferation. Hence, survivin expression is generally associated with poor prognosis [30,31]. The peptide ELTLGEFLKL can bind to HLA-A*0201, the most common human leukocyte antigen (HLA) serotype, and stimulate DCs to generate CD8^+^ immune responses against glioma cells [20-22,26]. Thus, the peptide was adopted in this study, mixed with GO at various feed ratios, and evaluated for its capability to induce a DC-mediated anti-glioma immune response.The amount of Ag loaded on GO nanosheets was assessed in this study. The Ag/GO feed ratios varied from 0.2 to 12.5. The Ag peptide and GO nanosheets were mixed under sonication for 30 min and then shaken for an additional hour. The mixtures were centrifuged and washed twice. The peptide amount in the supernatants was measured using a standard bicinchoninic acid (BCA) assay. As shown in Figure 1C, the amount of the Ag peptides that were loaded onto 1 μg GO increased from 0.18 μg to nearly 1 μg with increasing Ag/GO feed ratios. At the Ag/GO feed ratio of 3:1, the amount of peptide loaded on GO saturated at about 1 μg/1 μg.

**Figure 2:**
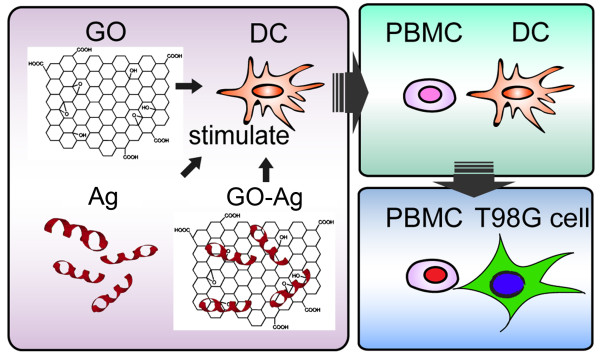
Schematic representation of the steps involved in DC-mediated anti-tumor immune response.

**Figure 3:**
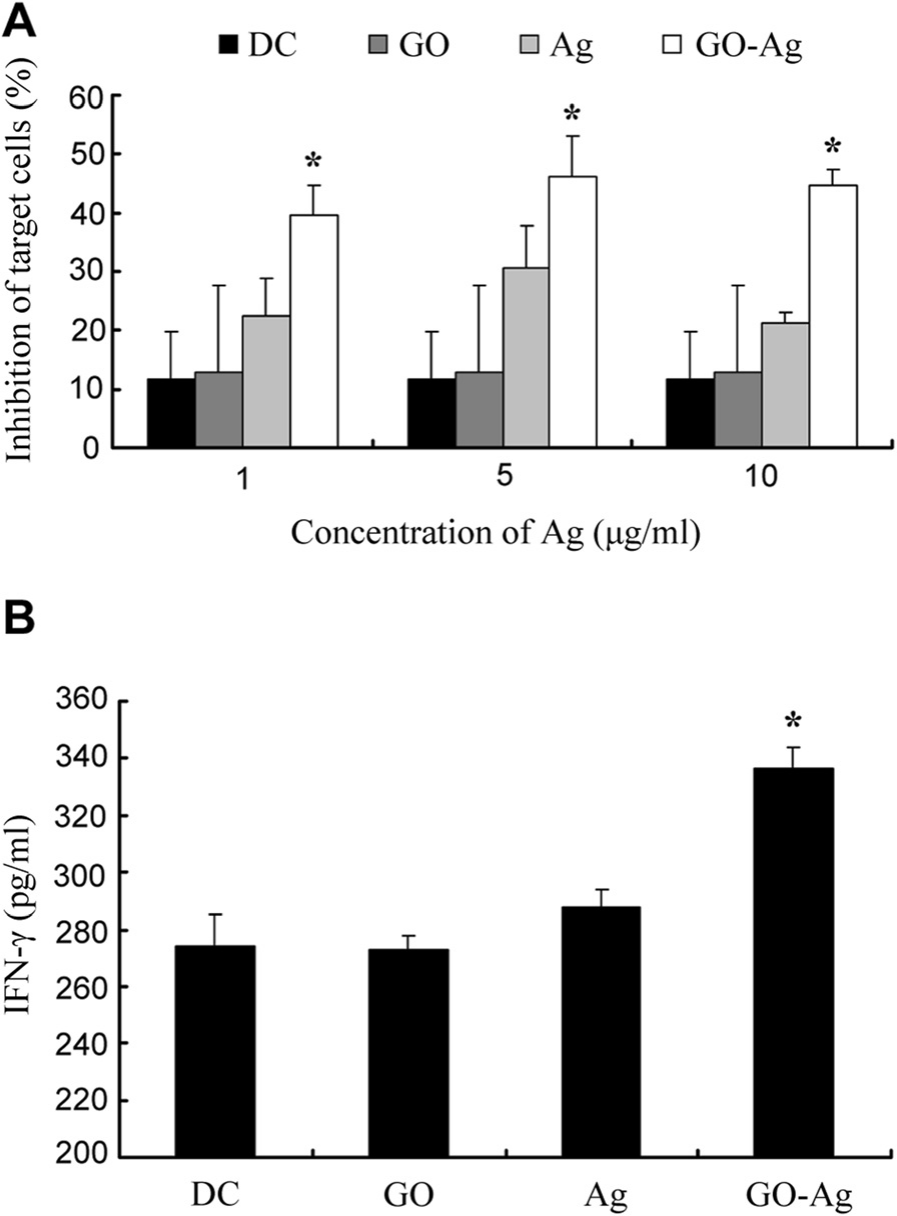
***In vitro *evaluation of the DC-mediated anti-tumor immune response.** DCs were treated with saline, GO, Ag, or GO-Ag. Treated DCs were mixed with PBMCs, which in turn were mixed with the target cells (T98G human glioma cell line) to elicit immune response. **(A)** Immune inhibition of glioma cells induced by un-pulsed, GO-pulsed, Ag-pulsed, or GO-Ag-pulsed DCs (mean ± standard deviation (std), *n* = 6). **(B)** IFN-γ secretion induced by un-pulsed, GO-pulsed, Ag-pulsed, or GO-Ag-pulsed DCs (mean ± std, *n* = 6). The stars indicate statistically significant differences among the groups.

We next evaluated whether GO would modulate the immunogenicity of the peptide antigen. The schematic representation of the steps involved is shown in Figure 2. A fixed concentration of GO (0.1 μg/mL) was mixed with Ag of various concentrations in the following experiments. The DCs were pulsed for 2 h with GO, Ag, or GO-Ag and co-incubated for 3 days with cognate peripheral blood mononuclear cells (PBMCs; serving as the effector cells), at the effector-to-target ratio (E:T) of 20:1. The PBMCs were subsequently co-incubated with the target glioma cells (T98G, human glioma cell line) for two more days, and the anti-glioma immune response was evaluated with a standard MTS assay [32]. The results were presented in Figure 3A. First, Ag-treated DC induced a higher anti-tumor response compared to un-pulsed DCs. For DCs pulsed with 1, 5, and 10 μg/mL of Ag, the corresponding tumor inhibition was 22%, 30.5%, and 21%, respectively. As a comparison, the inhibition induced by un-pulsed DCs was only 11.5%. Second, GO-Ag-treated DCs induced a significantly higher glioma inhibition compared to either Ag-treated or GO-treated DCs (Figure 3A, *p* < 0.05). For DCs treated with 1, 5, and 10 μg/mL of Ag mixed with GO, the corresponding inhibition rate was 39.5%, 46.5%, and 44.5%, respectively. It should be noted that 5 μg/mL of Ag triggered the highest anti-glioma response compared to the other concentrations, indicating that a proper amount of Ag was required for optimized anti-glioma reactions. As a result, in the following experiments, we used 5 μg/mL of Ag or GO-Ag to stimulate the DCs.

To verify the results of the above immune study, IFN-γ secretion was also measured in this work. IFN-γ is produced predominantly by T lymphocytes and plays a critical role in anti-tumor immunity. Hence, IFN-γ is commonly used as a surrogate indicator of anti-cancer immune responses [26]. DCs were pulsed and co-incubated with cognate PBMCs as described above. The IFN-γ in the supernatant was measured with standard ELISA. As shown in Figure 3B, GO-Ag treatment resulted in a significantly higher production of IFN-γ, again indicating that GO-Ag could trigger a more potent anti-glioma immune response compared with free Ag or GO alone.

**Figure 4:**
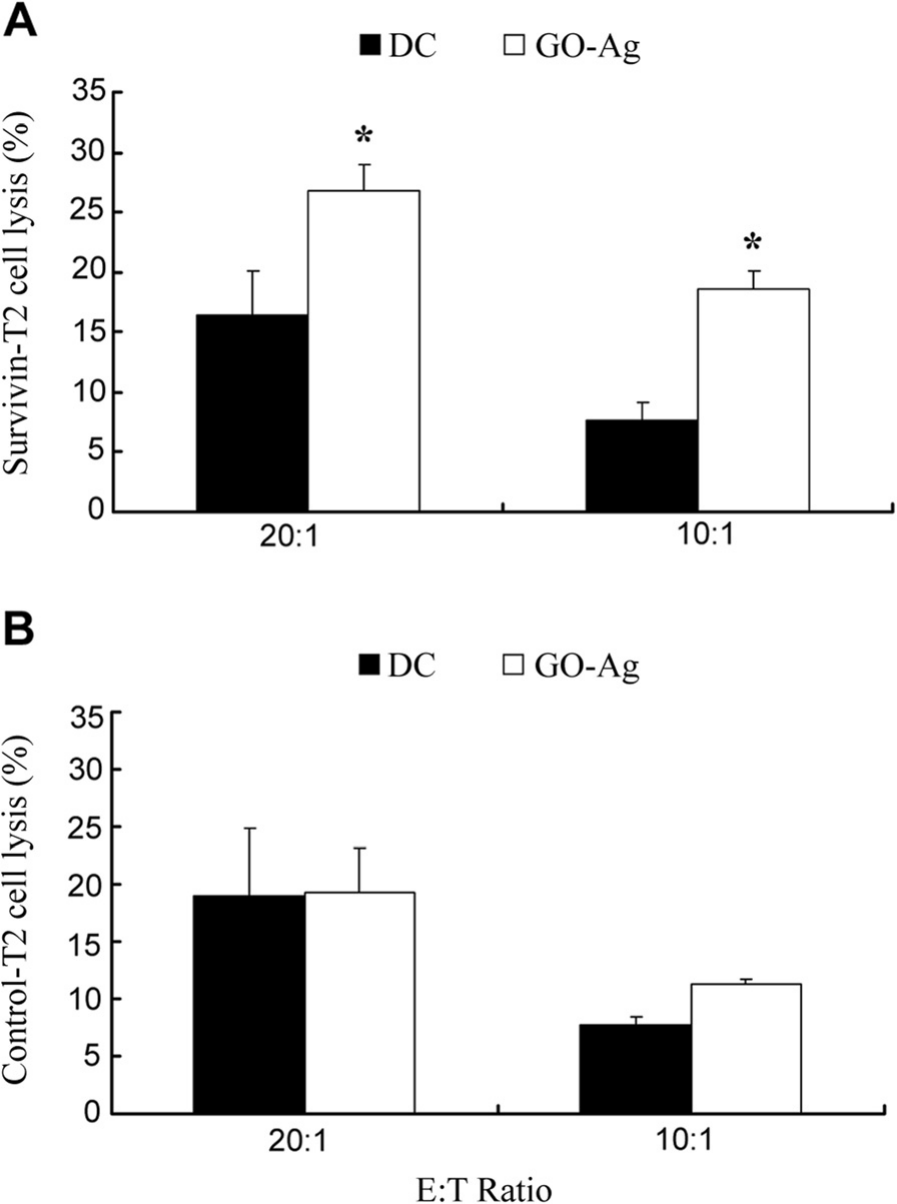
**Antigen-specific immune lysis of the target cells.** PBMCs were pretreated with un-pulsed DCs or GO-Ag-pulsed DCs. The treated PBMCs were co-incubated with either the Ag-loaded T2 cells **(A)** or the control peptide-loaded T2 cells **(B)** (mean ± std, *n* = 6). The stars indicate statistically significant differences between the groups.

The specificity of DC-mediated anti-cancer immune response is important due to concerns about autoimmune diseases. To evaluate whether the GO-Ag-enhanced immunity was specific for the Ag, DCs were pretreated with GO-Ag and co-incubated with PBMCs. The PBMCs were subsequently mixed with two types of target cells, T2 cells loaded with the Ag peptide (Ag-T2 cells) or T2 cells loaded with the control peptide APDTRPAPG (Control-T2 cells). Because T2 cells express HLA-A2 that can bind with the HLA-A2-restricted peptide, they are commonly used as model target cells for studying peptide-specific immune response [29]. Figure 4 reveals the immune study results. While GO-Ag significantly enhanced the immune response against Ag-T2 cells (Figure 4A), its effects on Control-T2 cells were minimal (Figure 4B). It could be deduced that, owing to the absence of Ag on the surfaces of Control-T2 cells, GO-Ag did not enhance the immunity against these cells. Thus, the GO-Ag-enhanced immunity was relatively specific towards the target cells carrying the Ag (survivin peptide) on the cell surface.

**Figure 5:**
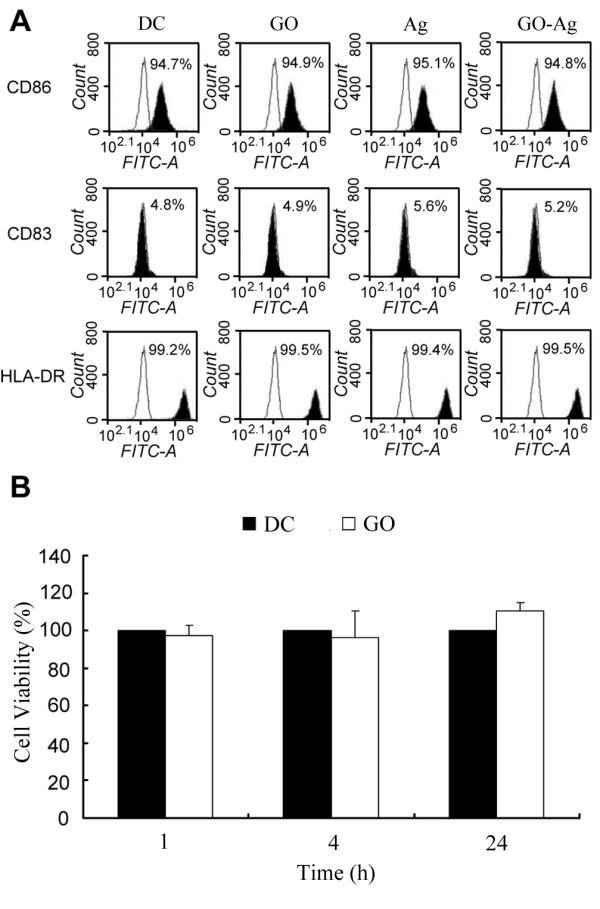
**Phenotype and cellular viability studies of the DCs after stimulation. (A)** Flow cytometry evaluation of CD86, CD83, and HLA-DR expression on DCs treated with GO, Ag, or GO-Ag. **(B)** Viability of DCs after being treated with 0.1 μg/mL of GO for 1, 4, or 24 h (mean ± std, *n* = 6).

The above results showed that GO could enhance the DC-mediated anti-glioma immunity. To explore the feasibility of using GO as an immune modulator in biomedical applications, it is important to investigate whether GO will affect the maturation and the viability of DCs. It is well known that DCs express multiple surface phenotype markers which are closely related to DCs' functions and maturation process [6,33,34]. In this work, we treated immature DCs with GO, Ag, or GO-Ag for 2 days and evaluated the expression of CD83, CD86, and HLA-DR on the DCs with antibodies and flow cytometry. Compared with the control, there was no significant difference in histogram profiles for DCs treated with GO, Ag, or GO-Ag (Figure 5A). The results suggested that GO or GO-Ag did not exert obvious adverse effects on the DC's maturation process. Next, we evaluated the toxicity of GO on human DCs. GO (0.1 μg/mL) were incubated with DCs for up to 24 h, and the viability of the cells was evaluated by the standard MTS assay. The results revealed no significant difference in the numbers of live cells between the GO-treated and control groups (Figure 5B). The data indicated that GO at the low concentration exhibited negligible toxicity against DCs, a result consistent with former toxicity studies of GO on Hela cells [35].

## Discussion

The aim of the study was to investigate whether a two-dimensional nanomaterial, GO, could be utilized to modulate DC-mediated anti-glioma immune reactions. The results showed that pulsing DCs with free Ag generated a limited anti-glioma response compared to un-pulsed DCs (Figure 3A). Pulsing DCs with GO alone failed to produce obvious modulation effects. However, stimulating DCs with GO-Ag significantly enhanced the anti-glioma immune reaction (*p* < 0.05), a finding that was further verified with the IFN-γ secretion experiments (Figure 3B). In addition, the enhanced immune response appeared to be relatively specific towards the target cells carrying the Ag peptide (Figure 4). Furthermore, at the concentration used in this study, GO exerted minimal toxicity to the DCs (Figure 5). These data suggested that GO might have application potential for enhancing the DC-mediated immune reactions against glioma cells.

The mechanisms of the observed immune enhancement are unclear at this stage. One hypothesis is that GO may serve as an immune adjuvant, which can activate the DCs and induce a more potent immune response. However, the data of this study showed that GO alone did not generate significant immune modulatory effects, a behavior inconsistent with most immune adjuvants (Figure 3A). Another possible mechanism is that GO may function as a carrier of the antigens for crossing the cell membrane [36] and thus bring more antigen into the DCs. Presumably more glioma antigens will be processed within the DCs, leading to an improved DC-mediated anti-glioma response. Obviously, extensive future studies are still warranted to unveil the immune-modulating mechanisms of GO.

The GO concentration used in this study was 0.1 μg/mL. At this concentration, we did not detect obvious GO toxicity against the DCs. This result was in agreement with prior toxicity studies of GO on Hela cells [35]. Interestingly, a recent study reported that high dosage of GO of 1 to 25 μg/mL suppressed antigen presentation in DCs and down-regulated the ability of DCs to activate antigen-specific T lymphocytes [37]. In comparison, the concentration of GO was orders of magnitude lower in our study, and the GO nanosheets were complexed with the antigens before interacting with the DCs. These differences highlight the importance of dosage and procedure of using GO, in that very different biological effects of GO may be generated depending on the experimental conditions.

## Conclusions

In summary, we observed that GO-Ag enhanced the DC-mediated anti-glioma immune response *in vitro*. Moreover, the immune response induced by GO-Ag appeared to be target-specific. Additionally, GO did not affect the viability or the phenotype of the DCs under our experimental conditions. These results indicated that GO might have potential utility for modulating DC-mediated anti-glioma immune reactions.

## Abbreviations

Ag: glioma peptide antigen; DCs: dendritic cells; GO: graphene oxide; GO-Ag: GO-Ag mixture; IFN-γ: interferon gamma; PBMCs: peripheral blood mononuclear cells.

## Competing interests

The authors declare that they have no competing interests.

## Authors’ contributions

WW carried out the immunoassays, participated in the design of the study, drafted the manuscript, and performed the statistical analysis. ZL carried out the materials study, participated in the design of the study, and drafted the manuscript. JD carried out the cell culture. CW and YF provided the graphene, participated in the design of the study, and helped to draft the manuscript. X-DY conceived of the study, participated in its design and coordination, and helped to draft the manuscript. All authors read and approved the final manuscript.
